# Stability of maxillary anterior teeth during retention and 1 year after removal of retention—an RCT on adolescents retained with two different bonded retainers and a vacuum-formed retainer

**DOI:** 10.1093/ejo/cjad020

**Published:** 2023-04-29

**Authors:** Sasan Naraghi, Niels Ganzer, Lars Bondemark, Mikael Sonesson

**Affiliations:** Orthodontic Clinic, Public Dental Health, Växjö, Sweden; Division of Orthodontics and Paediatric Dentistry, Department of Dental Medicine, Karolinska Institute, Stockholm, Sweden; Orthodontic Clinic, Public Dental Health, Gävle, Sweden; Centre for Research and Development Uppsala University/ Region Gävleborg, Gävle, Sweden; Department of Orthodontics, Faculty of Odontology, Malmő University, Malmő, Sweden; Department of Orthodontics, Faculty of Odontology, Malmő University, Malmő, Sweden

## Abstract

**Background:**

Maxillary bonded and removable retainers maintain teeth in correct positions following orthodontic treatment. There is insufficient evidence regarding the capacity of the retention methods to stabilize the maxillary teeth both during and after retention.

**Objective:**

To evaluate retention capacity and 1-year post-retention changes in the irregularity of maxillary anterior teeth and single anterior tooth contact point discrepancy (CPD) of two bonded and one removable retention method.

**Trial design:**

Three-arm parallel group single-centre randomized controlled trial.

**Methods:**

Ninety adolescent patients treated with fixed orthodontic appliances were enrolled. After gaining informed consent, the patients were randomized in blocks of 30 by an independent person into one of three groups: A) bonded retainer 13–23; B) bonded retainer 12–22; and C) removable vacuum-formed retainer. The primary outcomes were changes in Little’s irregularity index (LII) and single CPD measured on digitalized casts before retention (T1), after 2 years of retention (T2), and 1-year post-retention (T3).

**Blinding:**

The digital casts were blinded for the outcome assessor.

**Results:**

Data on all 90 patients were analysed according to intention-to-treat principles. Changes in LII during retention were 0.3 mm in group A, 0.6 mm in group B, and 1.0 mm in group C. No significant differences between the groups were seen (*P* > 0.05). Changes during post-retention were 1.1 mm in group A, 0.5 mm in group B, and 0.4 mm in group C. Group A showed more significant changes than groups B and C (*P* = 0.003). During the whole post-treatment period, no significant differences were shown between the groups (*P* > 0.05). CPD did not differ significantly between the groups at any point.

**Harms:**

Three patients showed changes of LII over 3 mm or CPD over 2 mm during the post-retention period, and two accepted to be realigned.

**Limitations:**

The trial was a single-centre study evaluating 1-year post-retention changes.

**Conclusions:**

The changes were clinically insignificant during and after the retention period. Thus, all three methods showed equal retention capacity.

**Trial registration:**

www.clinicaltrials.com (NCT04616755).

## Introduction

The stability of aligned teeth after orthodontic treatment is challenging, and relapse and natural changes may jeopardize the treatment results. Maintaining the position of the anterior maxillary teeth is of particular concern since this anatomical site, commonly defined as the aesthetic zone, is essential for patients when evaluating the outcomes after orthodontic treatment. In addition, relapse in this region is often why patients ask for re-treatment (1,2).

Several retention strategies for the maxilla are available. Usually, one of these three strategies is used: 1. a retainer bonded to the incisors and the canines, 2. a retainer bonded to the four incisors, or 3. a removable vacuum-formed retainer (VFR) covering the occlusal surfaces of all teeth (3). Bonded retainers have the advantage of being independent of patient compliance and are reported to have good retention capacity. The disadvantages are that the retainers frequently suffer from composite breakage or wire fractures. In addition, bonded retainers may also create difficulties in cleaning approximal sites, increasing plaque accumulation and gingival bleeding (3–7). However, this might not affect periodontal health (6). The most significant disadvantage of VFR is that patient compliance decreases over time (8–10).

Based on Reitan and co-workers’ histological findings (11), clinical praxis suggests that retainers should be used for approximately 1 year to stabilize the anatomical structure of the supracrestal periodontal fibres. However, the duration of wearing retainers is under discussion (12). The idea of ‘retentions are forever’ is based on trying to prevent or slow down the natural changes caused by ageing and has recently been adopted by many clinicians (10,13,14).

Most previous clinical follow-ups on stability during and after the retention period have mainly focussed on the mandibular anterior teeth. At the same time, only a few studies regarding the stability of maxillary anterior teeth seem to be available (10,15). Previously, a systematic review (16) showed that the retention capacity of bonded or removable retainers was equal in the maxilla during the first year of retention. However, the review included studies with a risk of bias with only low to moderate quality and still high-quality RCTs to produce evidence-based recommendations on retention procedures are requested (16,17). Thus, no firm conclusions could be drawn concerning the retention capacity of the different protocols. Furthermore, the crucial question of how stable the maxillary anterior teeth are after removing the retention devices remains unanswered.

This study examined the retention capacity and 1-year post-retention changes of the six maxillary anterior teeth when the retainers were removed. Both the increase in irregularity and single tooth contact point discrepancy (CPD) were evaluated for the three maxillary retention strategies: 1. bonded retainer to the six maxillary anterior teeth, 2. bonded retainer to the four maxillary incisors, and 3. a VFR covering all maxillary teeth. We hypothesized that post-retention changes would occur. However, even if significant, the overall post-retention changes would be clinically acceptable and equivalent between the retention groups and the changes seen during the retention period.

## Subjects and methods

### Participants

Initially, we invited patients with ongoing orthodontic treatment at the Orthodontic Clinic Växjö, Public Dental Service, Region Kronoberg, Sweden, to participate in the study. The inclusion criteria were adolescents treated with a fixed appliance in the maxilla or the maxilla and mandible. The exclusion criteria were patients with clefts or syndromes, patients with agenesis or extracted maxillary anterior teeth, patients treated with removable appliances and patients who underwent orthognathic surgery. Enrolment commenced in October 2013 and ended in October 2017. The patients were followed during a 2-year retention period, and retention devices were removed until October 2019. The patients were then further followed for a 1-year post-retention period until December 2020, when the stability of the maxillary anterior teeth was reassessed. The study was approved by the Regional Ethical Research Board, Linköping, which follows the Declaration of Helsinki (Dnr. 2013/131) and registered in Clinical Trials.gov (NCT04616755).

### Sample size calculation

The sample size was based on a clinically relevant and realistic mean difference of 3 mm (SD = 3 mm) for the Little’s irregularity index (LII) between the three retention groups described previously (18). The significance level was set to 5 per cent and the power to 90 per cent. The calculation resulted in a sample size of 24 patients per group.

### Trial design and outcomes

The study was a single-centre randomized controlled trial (RCT) with three parallel arms and a 1:1:1 allocation ratio.

Study casts were produced at four different time points: before treatment (T0); after removal of the fixed orthodontic appliances/start of retention (T1); after 2 years of retention when the retainers were removed (T2); and 1 year after the removal of the retainers (T3).

The primary outcomes were the post-treatment and post-retention changes in the irregularity of the six maxillary anterior teeth, according to LII, and a single CPD of 2 mm or more between T1, T2, and T3. Consequently, the changes in LII and CPD between the three retention devices were compared during both the retention period and the post-retention period.

The secondary outcomes were changes in arch length, intercanine, and intermolar width, maximum single tooth rotation of maxillary anterior teeth, and changes in overjet and overbite at T1, T2, and T3.

### Measurements

**Figure 1:**
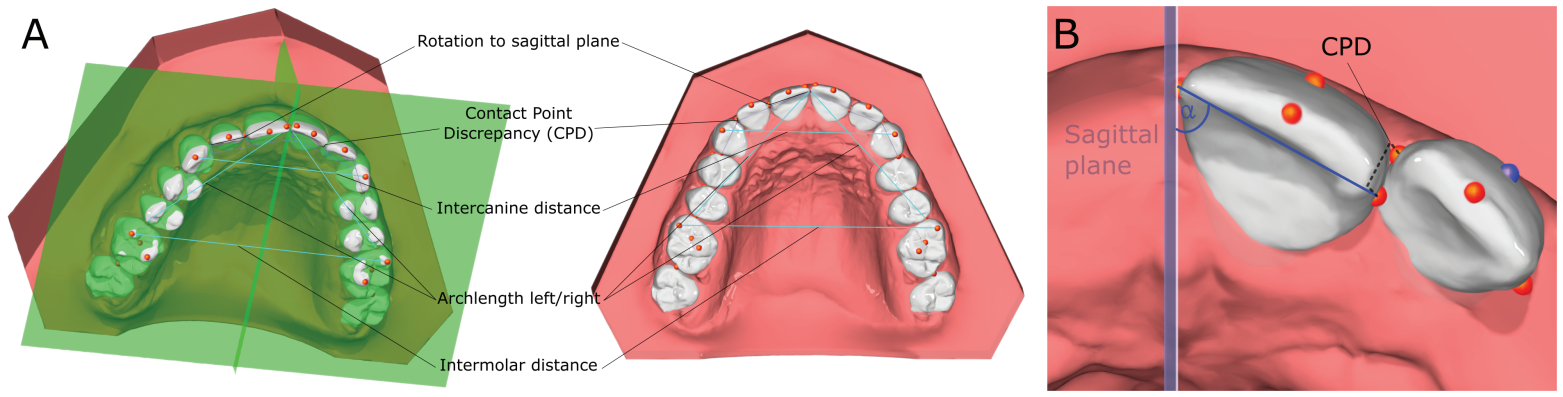
(A) Primary and secondary outcome measures in OnyxCeph^3^™. (B) Contact point discrepancy (CPD) and rotation angle (alpha) between a line through the tooth’s mesial and distal measurement point and a sagittal plane.

Digitalized three-dimensional (3D) study casts were produced for measurements of the three retention groups before treatment (T0), at the end of active treatment/start of retention (T1), after the 2-year retention period (T2) and 1 year after the post-retention period (T3). Before the assessment of measurements, the study casts were digitized with a stationary 3D scanner (D3, 3Shape, Copenhagen, Denmark). The measurement points were located on the digital models using OnyxCeph^3^™ software (v3.2.142, Image Instruments, Chemnitz, Germany) with semi-automatic segmentation. The measurement points were manually adjusted to improve consistency. The measurement points for CPD were defined as the mesial and distal points of the broadest mesiodistal diameter of the tooth. Further, the CPD was measured as a projection on the occlusal plane (Figure 1A and 1B).

### Randomization

The patients were asked to participate in the trial 3 months before the removal of the fixed appliances, as described in an earlier publication (18). After informed consent was gained from the patients and their custodians, the participants were randomized into one of the three retention groups: A) bonded retainer 13–23; B) bonded retainer 12–22; and C) removable VFR covering the maxillary teeth, including the second molars. An independent person not involved in the trial and analysis prepared the randomization, which was then carried out by three staff members not involved in the trial. A block of 30 notes, 3 × 10 separately sealed small envelopes of each retention method, were placed in three sealed opaque large envelopes. Every participant picked an envelope and revealed the random group assignment by opening the envelope. Recruitment continued until the total number of estimated subjects was achieved.

### Intention-to-treat

All randomized patients were included in the final statistical analyses. Patients who moved from the area or did not accept the removal of the retentions were still included in the statistical analysis by imputing the variables’ mean values for the allocated group. Thus, all 90 patients (30 per group) could be included in all trial analyses.

### Blinding

Due to the trial’s design, neither the participant nor the operator could be blinded for the group assignment. However, the assessor (NG) was blinded since impressions were taken after removing the retention appliance, and the study casts were anonymised.

### Clinical procedure

During the orthodontic treatment, three experienced orthodontists treated the patients with a pre-adjusted fixed appliance in the maxilla and mandible (0.022 slot size, MBT prescription, Victory Series, 3M Unitek, Monrovia, California, USA). After removing the fixed appliances, post-treatment records, including study casts, were taken (T1). After 2 years of retention, the patients were asked to approve the removal of the retention appliances. Post-retention records and study casts were then taken (T2). All patients in group C handed in the VFR to the clinic after the retention period. One year after the removal of the retainers, additional records and study casts were taken (T3).

### Retention methods

**Figure 2:**
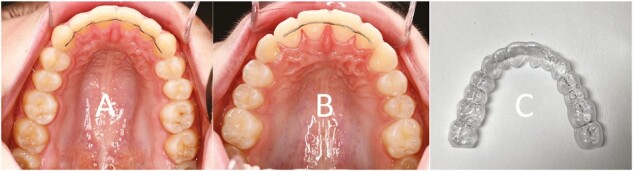
(A) Bonded retainer 13–23. (B) Bonded retainer 12–22. (C) Vacuum-formed retainer 17–27.

The retainers in group A (13–23, Figure 2A), group B (12–22, Figure 2B) and Group C (Figure 2C) were manufactured as described in an earlier publication (18). The patients in group C followed a standard prescription for VFR wear: 22–24 hours/day during the first 4 weeks, then every night. The wearing time was reduced 1 year after debonding to every other night.

### Statistical analysis

All data were processed with R (v. 4.2.2) (19). The primary and secondary outcome measures were tested for normality in distribution with histograms and the Shapiro–Wilk test. Variance homogeneity was tested with Levene’s test. Since almost all variables showed non-normal distribution, the Kruskal–Wallis test was used for a three-group comparison of continuous variables, followed by Dunn’s test. Holm–Bonferroni correction was applied to compensate for multiple comparisons. Linear multiple regression analysis was used to test possible predictors for change in irregularity during the post-retention period. Differences with a *P*-value less than 5 per cent (*P* < 0.05) were considered statistically significant.

### Method error analysis

One assessor (NG) measured repeated segmentations and measurements on 15 randomly selected cases after at least 2 weeks. The two-measurement series were compared using the Bland–Altman method (20). Moreover, a paired *t*-test was used to disclose if any significant differences existed between the two-measurement series, that is, to test whether there were systematic errors in the measurements.

## Results

### Sample characteristics

**Figure 3:**
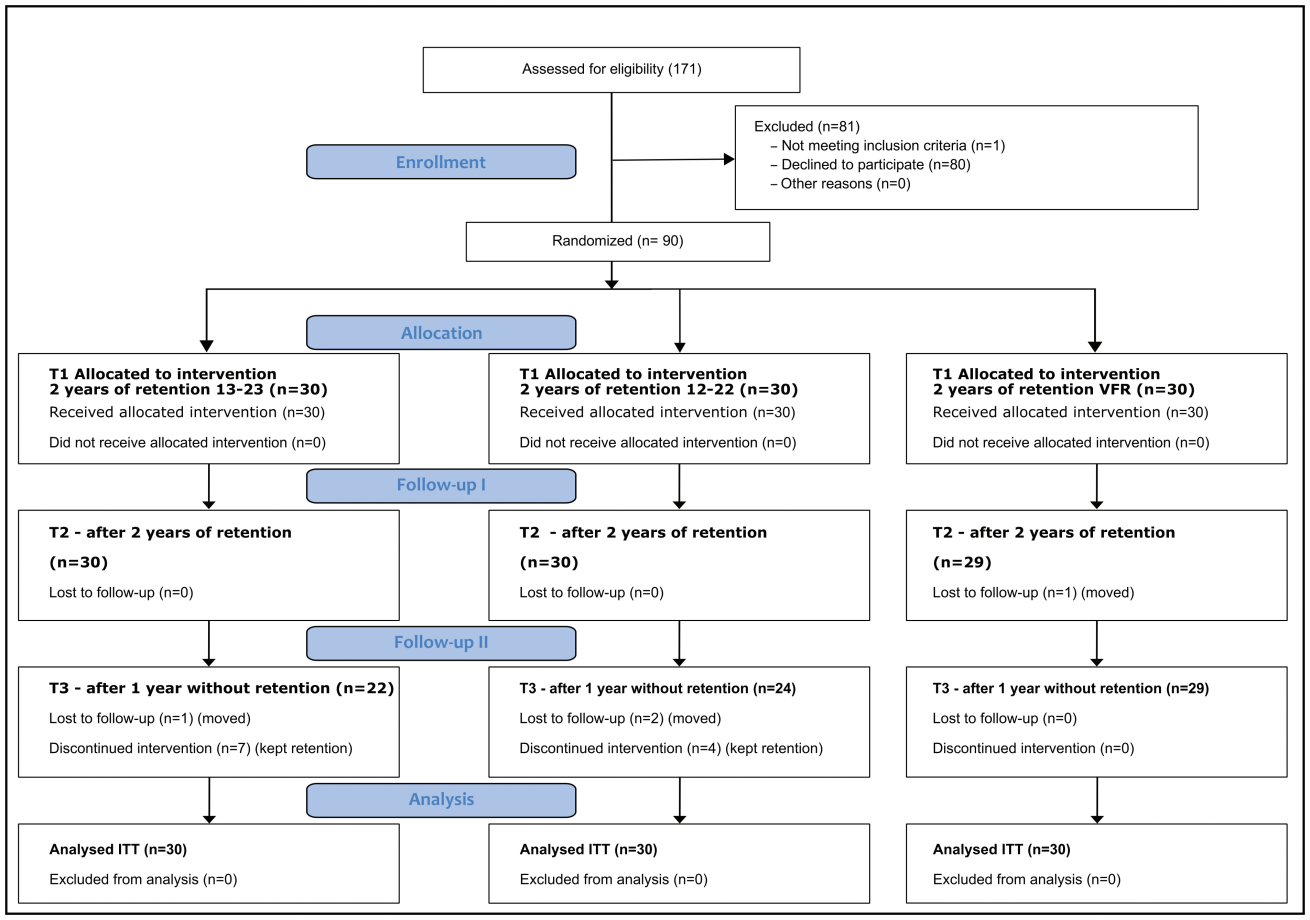
Consort flow chart.

Initially, 90 patients (54 females and 36 males) with a mean age of 13.9 years agreed to participate in the trial. After 2 years of retention, all the patients were asked to remove the retainers in the maxilla. Eleven patients refused removal: seven in group A (bonded retainer 13–23) and four in group B (bonded retainer 12–22). Further, one patient in group A, two in group B, and one in group C (VFR) moved during the 1-year follow-up. According to the Intention-to-treat (ITT) principle, all patients who kept the retainers or moved location remained within their allocated groups. Figure 3 shows the trial’s flowchart. Demographic data at the start of treatment (T0), at debonding/start of retention (T1), 2 years with retention (T2) and one year after removal of retention (T3) is shown in Table 1. There were no statistically significant differences at T0 concerning age, gender, rotations, maximum CPD, extractions or duration of treatment between the three groups.

**Table 1.** Baseline demographic data. *P*-values calculated with Kruskal–Wallis test for continuous variables and chi-square test for categorical variables

Group	*n*	Pre Tx Age, yrs mean (SD)	Max rotation T1−T0 mean (SD)	Extractions, *n*	Bimaxillary Tx, *n*	Tx duration, months mean (SD)
A—Bonded retainer 13–23	30 (♀ 17; ♂ 13)	13.8 (1.5)	11.8° (8.0)	12	23	24.3 (10.4)
B—Bonded retainer 12–22	30 (♀ 20; ♂10)	14.0 (1.8)	14.1° (10.6)	11	25	22.3 (8.3)
C—Vacuum-formed retainer	30 (♀ 17; ♂ 13)	13.9 (1.9)	14.3° (9.5)	5	17	24.6 (12.9)
*P* 3-group-comparison	−0.659	0.893	0.520	0.108	0.056	0.852
Total population	90 (♀ 54; ♂ 36)	13.9 (1.7)	13.4° (9.4)	28	65	23.7 (10.6)

### Method error analysis

The method analysis confirmed an excellent agreement and low bias for all variables. Further, the paired *t*-tests did not reveal any statistically significant differences between the two-measurement series. Thus, there were no significant systematic errors in the measurements. The absolute mean measurement error for CPDs was 0.1 mm, for LII 0.2 mm, for overjet, overbite, arch length, intercanine, and intermolar width 0.1 mm each and 2.3 degrees for tooth rotations.

### Changes in primary outcomes

**Figure 4:**
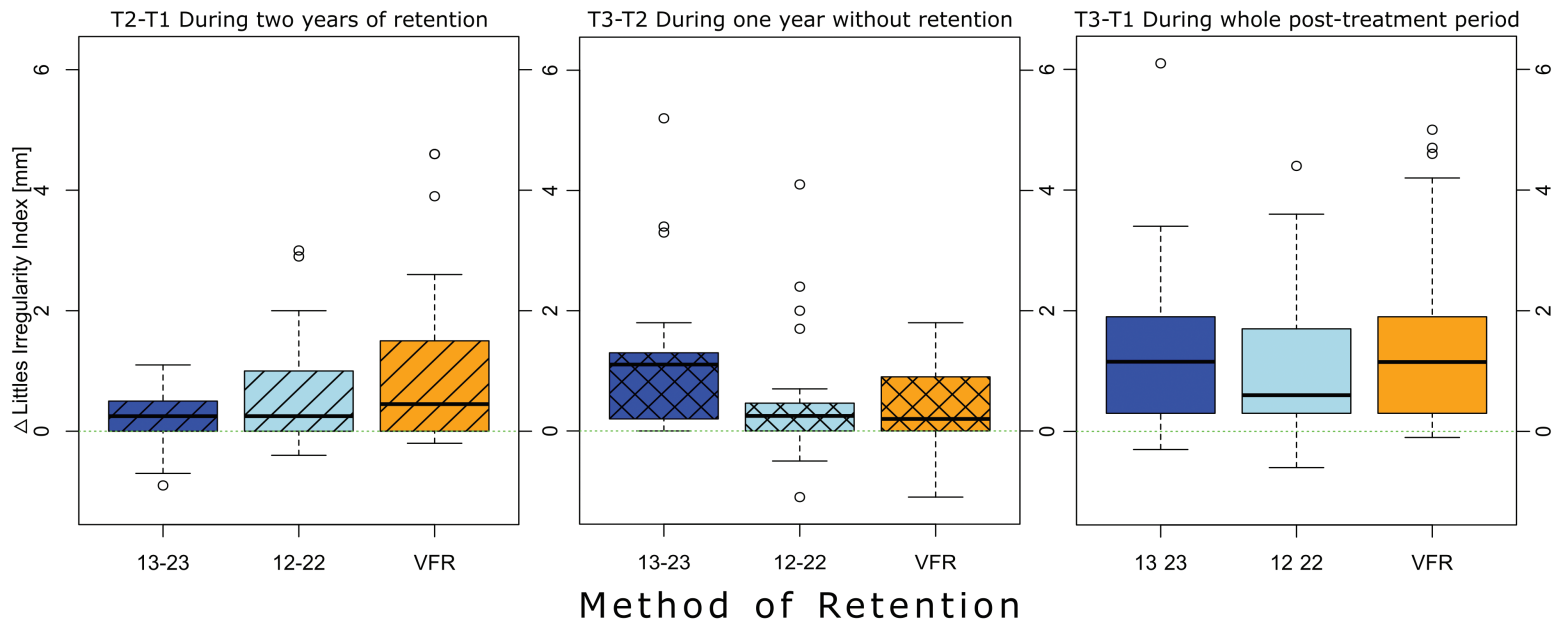
Boxplots showing the changes in Little’s irregularity index (mm) during the 2-year retention period (T2–T1), followed by 1 year without retention (T3–T2) and the whole post-treatment period (T3–T1). Group A: bonded retainer 13–23 (blue), Group B: bonded retainer 12–22 (light blue), Group C: vacuum-formed retainer (orange).

Retention, post-retention and post-treatment changes are summarized with means and 95 per cent confidence intervals in Table 2. Further, the changes in LII are depicted as Tukey boxplots in Figure 4. Regarding the whole post-treatment period of 3 years (T3–T1), mean changes in LII were 1.4 mm in group A (bonded retainer 13–23), 1.0 mm in group B (bonded retainer 12–22), and 1.4 mm in group C (VFR). Differences between groups were not statistically significant (*P* = 0.703).

**Table 2.** Retention (T2−T1), post-retention (T3−T2) and post-treatment (T3−T1) changes. Results on intention-to-treat basis represented by means and 95 per cent confidence intervals (CI). Tests were conducted with Kruskal–Wallis test ^a^ for 3-group comparison followed by Dunn´s test ^b^ for between-group comparison. *P*-values adjusted for multiple comparisons with Holm–Bonferroni correction. T2 = after 2 years of retention, T3 = after 1 year without any retention

			A Bonded retainer 13-23 (n=30)			13-23 vs. 12-22	B Bonded retainer 12-22 (n=30)			12-22 vs. VFR	C VFR (n=30)			VFR vs. 13-23
		3-group comparison	Mean	95% CI			Mean	95% CI			Mean	95% CI		
		*P* ^*a*^		lower	upper	*P* ^b^		lower	upper	*P* ^b^		lower	upper	*P* ^b^
	LII (T2-T1)	.134	0.3 mm	0.1	0.5	.397	0.6 mm	0.4	0.8	.500	1.0 mm	0.4	1.6	.138
Change T3-T2	LII	.003	1.1 mm	0.7	1.5	.007	0.5 mm	0.1	0.9	.890	0.4 mm	0.2	0.6	.007
	Arch length	.044	0.1 mm	−0.1	0.3	.285	0.2 mm	0.0	0.4	.039	0.0 mm	−0.2	0.2	.307
	Intercanine width	.498	0.0 mm	−0.2	0.2	.782	0.0 mm	−0.4	0.4	.772	0.0 mm	−0.2	0.2	.784
	Intermolar width	.433	0.1 mm	−0.1	0.3	.725	0.1 mm	−0.1	0.3	.915	0.2 mm	0.0	0.4	.575
	Overjet	.031	−0.3 mm	−0.5	−0.1	.051	−0.1 mm	−0.3	0.1	.933	−0.1 mm	−0.3	0.1	.061
	Overbite	.033	0.4 mm	0.2	0.6	.570	0.4 mm	0.2	0.6	.108	0.2 mm	0.0	0.4	.038
	Maximum rotation	.095	16.1°	12.2	20.0	.364	3.5°	2.7	4.3	.094	4.5°	3.7	5.3	.415
	LII (T3-T1)	.520	1.4 mm	1.0	1.8	.792	1.0 mm	0.6	1.4	.887	1.4 mm	0.8	2.0	.726
	Max CPD T1	.149	0.5 mm	0.3	0.7	.497	0.4 mm	0.2	0.6	.431	0.4 mm	0.4	0.4	.157
	Max CPD T2	.520	0.6 mm	0.6	0.6	.653	0.8 mm	0.6	1.0	.951	0.8 mm	0.6	1.0	.984
	Max CPD T3	.619	1.0 mm	0.8	1.2	.999	0.9 mm	0.7	1.1	.949	1.0 mm	0.8	1.2	.830

Changes in LII during the 2-year retention period (T2–T1) were of non-significant magnitude (*P* = 0.134). The mean value for changes in LII in group A was 0.3 mm, in group B 0.6 mm, and group C 1.0 mm.

The changes during the 1-year post-retention period (T3–T2) were 1.1 mm in group A, 0.5 mm in group B, and 0.4 mm in group C. According to the Kruskal–Wallis test results, there was a statistically significant difference between the three groups (*P* = 0.003). Dunn’s test revealed that group A showed statistically significant and more LII changes than group B (*P* = 0.007) and C (*P* = 0.007).

The maximum CPD (Max CPD) mean showed no statistically significant differences. At T3, Max CPD was 1.0 mm in group A, 0.9 mm in group B, and 1.0 mm in group C.

Linear multiple regression analysis was used to test possible predictors. However, no good prediction model could be found using the maximum pre-treatment CPD, the maximum derotation during treatment, and the maximum CPD at debonding, age and gender.

### Changes in secondary outcomes

During the post-retention period (T3–T2), arch length increased by 0.2 mm in group B compared to 0.0 mm in group C (*P* = 0.039). Likewise, overbite increased by 0.2 mm in group C compared to 0.4 mm in group A (*P* = 0.038). Also, the comparison of overjet indicated statistically significant differences (*P* = 0.031) during this period. However, Dunn’s test revealed no other significant differences when applying the Holm–Bonferroni correction. Likewise, the other secondary outcomes did not show statistically significant differences (Table 2).

### Harms

During the 1-year post-retention period, three patients, two in group A (bonded retainer 13–23) and one in group B (bonded retainer 12–22) developed irregularities greater than 3 mm for the maxillary anterior teeth or CPD greater than 2 mm. Two of the three patients accepted to be realigned.

## Discussion

### Main findings

In this trial, we evaluated the stability of the maxillary anterior teeth during two years of retention followed by 1 year without retention. We found minor changes in Little’s irregularity index during these post-treatment periods. After the retention was removed, small but significant mean differences were found between group A (bonded retainer 13–23) and groups B (bonded retainer 12–22), and C (VFR) (1.1 mm versus 0.5 and 0.4 mm, respectively). However, group A had the slightest changes in LII during the 2-year retention period. During the whole 3-year post-treatment time (T3–T1), small and clinically insignificant group differences were found (1.0 to 1.4 mm) (Table 2).

For the secondary outcomes of arch length and overbite, statistically significant changes between the groups were observed (Table 2). Moreover, we could verify that the changes during and after retention were small and clinically acceptable. Thus, our initial hypotheses that there should be no statistically significant differences between the groups were confirmed and that the overall post-retention changes would be clinically acceptable. On the other hand, every retention group comprised a few patients with an increase in the irregularity of more than 3 mm. These patients are depicted as outliers in Figure 3. In Group C (VFR), the most considerable changes in irregularity were assessed during the retention period; in group A (bonded retainer 13–23), the most considerable changes were measured during the post-retention period.

### Interpretations

It was evident that even if the anterior teeth were retained for 2 years, small short-term changes occurred during the retention period and, of course, during the 1-year post-retention period. The initial benefits from the bonded retainer 13–23 in group A during the retention period were equalised after removing the retention appliance. This finding contrasts with the assumption that most relapse occurs during the first years after debonding the orthodontic appliance. However, when the bonded retention appliance was removed after 2 years, there were some immediate changes, particularly in group A. Unfortunately, no predictors for the increase in irregularity could be identified. Since the sample size was not calculated with multiple regression analysis in mind and the changes were minor, this trial might have too little power for this type of analysis.

Anyway, the knowledge from this trial about changes during the first post-retention year is essential and hypothesis-generating for further investigations of post-treatment stability. In the 1980s and 1990s, 1 to 3 years retention periods were considered adequate. Then, the 20-year follow-up published by Little *et al*. (21). initiated a paradigm shift towards lifelong retention to avoid changes in mandibular anterior alignment. In recent years, also the patients demand of perfectly aligned teeth have resulted in a lifelong retention approach, not only to prevent relapse after orthodontic treatment, but to avoid and prevent natural changes as well. In this light, removing the retention appliances might seem provocative, and in that perspective, makes this trial unique. However, we believe and today can witness that the pendulum partly swings back since there are multiple reasons why lifelong retention may have drawbacks. Concerning removable retention appliances, long-term compliance obviously can be a problem. Krämer *et al*. (10). recently reported that 72 per cent of the patients ceased to use or seldom wore the recommended VFR after 5 years. Therefore, most patients with VFR can be regarded as ‘post-retention’ despite our recommendation to use the VFR some nights per week as long-term retention. Even fixed retainers have known issues, such as breakage and detachments. More importantly, Katsaros *et al*. (22). showed unexpected torque expressions and gingival recessions correlated to the use of fixed retainers bonded to the anterior teeth of the mandible.

However, among our patients´ parents, we frequently meet those who did not need lifelong retention. Even if treated decades ago with only a few years with retention, we still find almost perfectly aligned teeth. Thus, long-term retention should be recommended only for those who need it. In this context, it seems promising that the total increase in irregularity shown for all three groups in this trial was minor. Nevertheless, when advising the patient on further use of the retention or not, it is necessary to listen to the patient´s demand and inform about the risk of changes of the maxillary anterior teeth alignment.

### Strengths

This study explored a sample representative of patients at a specialist clinic in orthodontics regarding treatment indication, age and gender distribution. The trial used a robust randomized design with concealed allocation. Consequently, there was a low risk of selection bias. Furthermore, the risk of detection bias was low since the assessor was blinded for group assignment. Moreover, primary and secondary outcome measures were assessed using valid and reliable methods (23).

### Limitations and generalisability

This single-centre RCT has limited generalisability compared to a multi-centre RCT since the latter usually has more involved staff members and operators, which can decrease the risk of bias. Furthermore, it is reported that single-centre trials tend to report higher treatment effects than multi-centre studies (24).

This study presented some dropouts. A per-protocol analysis would consequently suffer from attrition bias. Thus, we applied the ITT principle and replaced the missing values with the group’s mean. This imputation may impact the results, and no golden rule for the imputation exists. Instead, the choice of imputation method must be evaluated for every trial. It can be discussed whether other methods of imputation or per-protocol analysis would have been preferable. However, as Nich and Carroll (25) described, authors should try to use ITT analysis in RCTs. Commonly missing values represent non-compliant participants with the ‘worse’ values for specific variables. In the present trial, four participants moved, and seven decided to continue with their retention appliance. The participants who moved were assumed to be comparable to an average participant. However, the participants continuing with the retention would reasonably present more stable results than an average participant. Thus, the imputation of the group means for the missing values results in maintained statistical power and adequate estimation of the outcomes.

Finally, a long-term evaluation of the stability of the maxillary incisors is necessary. Nonetheless, the present short-term evaluation provides essential information for a long-term follow-up. Also, it supports the results of a previous retrospective 10-year follow-up, which shows that the most considerable changes occur in the first and second year after retention (26). Moreover, it may be unethical to start with a long-term follow-up evaluation before data about changes in the short run is available. Thus, it is highly motivated to perform a short-term evaluation initially.

## Conclusions

All three retention strategies, a bonded retainer 13–23, a bonded retainer 12–22 and VFR, showed similar effectiveness in maintaining stability 1 year after the removal of the retention appliances. Even if minor changes occurred continuously, the overall increase in irregularity was clinically acceptable. However, from a stability perspective, a follow-up during 1 year is relatively short. Therefore, further evaluation of long-term stability is needed to make well-founded patient-care decisions.

## Data Availability

The data connected to the present article were provided by Region Kronoberg, Sweden, under licence/by permission. Data will be shared on request to the corresponding author with the approval of Region Kronoberg, Sweden.

## Trial registration

www.clinicaltrials.com (NCT04616755).
